# Rift Valley Fever and Crimean-Congo Hemorrhagic Fever Viruses in Ruminants, Jordan

**DOI:** 10.3201/eid2702.203713

**Published:** 2021-02

**Authors:** Mohammad M. Obaidat, James C. Graziano, Maria Morales-Betoulle, Shelley M. Brown, Cheng-Feng Chiang, John D. Klena

**Affiliations:** Jordan University of Science and Technology, Irbid, Jordan (M.M. Obaidat);; Centers for Disease Control and Prevention, Atlanta, Georgia, USA (J.C. Graziano, M. Morales-Betoulle, S.M. Brown, C.-F. Chiang, J.D. Klena)

**Keywords:** epidemiology, viruses, Rift valley fever, Crimean-Congo hemorrhagic fever, zoonoses, ruminants, Jordan, Middle East, vector-borne infections

## Abstract

The epidemiology of Rift Valley fever virus (RVFV) and Crimean-Congo hemorrhagic fever virus (CCHFV) in Jordan is unknown. Our investigation showed 3% of 989 tested dairy cattle, sheep, and goats were RVFV seropositive and 14% were CCHFV seropositive. Ongoing surveillance is needed to assess risk to humans and protect public health.

Rift Valley fever (RVF) virus (RVFV) and Crimean-Congo hemorrhagic fever (CCHF) virus (CCHFV) are zoonotic arboviruses. RVFV has been causing sporadic outbreaks in East, West, and southern Africa; the Indian Ocean region; and the Arabian Peninsula (Saudi Arabia and Yemen) (*1*). Although Jordan is considered an at-risk country, the disease has not been reported in Jordan (*2*). Meanwhile, no seroprevalence studies for CCHFV in human or animals have been conducted in Jordan despite the endemicity of CCHF in neighboring countries (https://www.cdc.gov/vhf/crimean-congo/outbreaks/distribution-map.html), the presence of a necessary tick vector (*Hyalomma* sp.) (https://www.who.int/csr/disease/crimean_congoHF), and the classification of Jordan as an at-risk country (*3*). Accordingly, we aimed to determine whether livestock populations across Jordan have been exposed to CCHFV and RVFV (Appendix). Jordan University of Science and Technology Animal Care and Use Committee approved the study. 

Using EpiTool (https://epitools.ausvet.com.au), we determined that a minimum of 665 samples were required based on an assumed prevalence of 0.5% and a 95% CI. We tested 989 serum samples from 109 farms (31 dairy cow farms, 44 sheep farms, and 20 goat farms, as well as 14 mixed sheep and goat farms) that were randomly selected from different regions of Jordan during 2015–2016. Serum samples were shipped to the US Centers for Disease Control and Prevention (Atlanta, Georgia USA) for laboratory testing by indirect ELISA (Appendix).

Overall seroprevalence was 14% for CCHFV and 3% for RVFV. The greatest differences in seroprevalence were among sheep, 16.7% (85/509) for CCHFV and 4.5% (23/509) for RVFV, followed by a similar difference for goats, 14.7% (48/327) for CCHFV and 0.6% (2/327) for RVFV (Table). CCHFV and RVFV seroprevalence did not differ in cows at ≈1% (4/152 for CCHF and 2/152 for RVF) (Table).

**Table:** Seroprevalence of CCHFV and RVFV by location and animal species, Jordan, 2015–2016*

Location	Seroprevalence, %														
	Sheep				Goat				Cow				All animals		
	No. tested	CCHFV	RVFV		No. tested	CCHFV	RVFV		No. tested	CCHFV	RVFV		No. tested	CCHFV	RVFV
Ajloun	36	80.5	5.6		42	85.7	0		0				78	83	2.6
Zarqa (Al-Dulail area)	0	NA	NA		0	NA	0		100	0	2		100	0	2
Amman	0				12	0	0		3	0	0		15	0	0
Irbid and Northern Jordan Valley	206	16.5	1.4		39	2.6	0		26	15.4	0		271	14.4	0.7
Jarash	78	8	0		127	4	0		10	0	0		215	5	0
Karak	8	0	0		24	0	4		1	0	0		33	0	3
Ma’an	12	8	0		13	0	0		0				25	5	0
Mafraq	94	5	13		42	5	0		8	0	0		144	5	8
Balqa	16	0	0		7	43	14		4	0	0		27	11	4
Tafilah	59	17	10		22	0	0		0				81	13	8
Total	509	16.7	4.5		328	14.7	0.6		152	2.6	1.3		989	14	3

*CCHFV, Crimean-Congo hemorrhagic fever virus; RVFV, Rift Valley fever virus.

The provinces that had the highest respective seroprevalence for CCHFV or RVFV did not coincide (Figure). The highest CCHFV seroprevalence was found in the northwest and the highest RVFV seroprevalence in the provinces along the central western border area with Israel (Figure). In total, 29 farms had seropositivity for CCHFV: 19 sheep farms (10 in Irbid, 5 in Tafela, 2 in Jarash, 1 in Ma’an, and 1 in Mafraq), 5 mixed sheep and goat farms (1 in each of Irbid, Jarash, Ajloun, Mafraq, and Balqa), 3 goat farms (all in Jarash), and 2 dairy cow farms in Irbid. Ten farms had animals seropositive for RVFV: 5 sheep farms (2 in Tafelah, 2 in Irbid, and 1 in Mafraq), 3 mixed sheep and goat farms (1 in each of Ajloun, Mafraq, and Balqa), 1 goat farm in Karak, and 1 dairy-cow farm in Zarqa.

**Figure:**
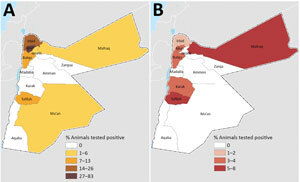
Seroprevalence of Crimean-Congo hemorrhagic fever (A) and Rift Valley fever (B) in ruminants, by province, Jordan, 2015–2016.

This study reports RVFV seropositivity in Jordan’s ruminant population without any previously reported animal cases. Observing seropositive animals without disease, however, is not unique; 22% of the small ruminant population in Mayotte were seropositive (*4*) without any documented human or animal clinical cases. Similarly, South Africa reported high proportion of seropositive ruminants in the absence of a reported outbreak (*5*). In addition, IgG seroprevalence of 6.5% was detected in sheep and goats in southern Gabon without a reported outbreak (*6*).

In Jordan, small ruminants are short day breeders; June–September are breeding months. After a ≈5-month gestation period, lambing occurs during November–February, which places gestation and lambing periods during the rainy months in Jordan. The shift of RVF from enzootic to epizootic or epidemic cycle typically follows extended periods of heavy rainfall (*7*). Because rainy season and gestation periods overlap, RVFV spread poses a potential high risk for abortions and neonatal death in Jordan.

In light of the regional distribution and general expansion of RVFV and CCHFV into newly identified areas, it is not surprising that animals in Jordan tested seropositive to either virus. This finding is consistent with recent studies that reported other mosquitoborne viruses in Jordan, such as West Nile (*8*) and dengue viruses (*9*), and tickborne viruses such as *Coxiella burnetii* (*10*).

The findings of seropositive animals for CCHFV and RVFV in different regions of Jordan call for implementing an early warning contingency plan. Such a plan would include training field veterinary officers, developing strong epidemiologic capabilities, sustaining active disease surveillance, and enhancing laboratory diagnostic capabilities. On the basis of on our identification of the subprovinces with the highest seroprevalence, small ruminant sentinel herds should be monitored for IgG and IgM to these viruses in conjunction with seasonal weather, particularly before and during the rainy months. Despite CCHF virulence in humans and the potential public health impact because of severe outbreaks, the virus is not pathogenic for the amplifying hosts (i.e., ruminants). Thus, farmers and veterinarians are at higher risk for infection compared with the general population. Future studies should be conducted to determine the prevalence and potential incident cases of CCHF and RVF in Jordan’s human and animal populations. Ongoing surveillance will inform contemporaneous risk assessments and enable development of effective public health messaging for identified risk groups. 

Appendix Additional information about Crimean-Congo hemorrhagic fever and Rift Valley fever viruses in ruminants, Jordan, 2015–2016.
