# Supplementary material for: Rift Valley Fever and Crimean-Congo Hemorrhagic Fever Viruses in Ruminants, Jordan
Source: Emerg Infect Dis. 2021 Feb;27(2):653–5. doi: 10.3201/eid2702.203713 (PMC7853544; doi:10.3201/eid2702.203713)
Supplement: Appendix — Additional information about Crimean-Congo hemorrhagic fever and Rift Valley fever viruses in ruminants, Jordan, 2015–2016. [file 20-3713-Techapp-s1.pdf]

Article DOI: https://doi.org/10.3201/eid2702.203713

Rift Valley Fever and Crimean-Congo Hemorrhagic Fever Viruses in Ruminants, Jordan

Appendix

Dairy Ruminant Population in Jordan

The population of dairy cattle and small ruminants such as sheep and goats in Jordan was discussed in previous publications (*1*,*2*). Briefly, Jordan relies on dairy ruminants to meet the local demand on milk and dairy products, so ruminants are an important component of the Jordanian animal industry (*3*). Jordan has 2 dairy cattle production systems, both of which raise Holstein-Friesian dairy cows (*3*): a large intensive production system located in Al-Dulial area that uses modern management practices and accounts for ≈50% of the milk in Jordan (*3*), and a small-scale production system that uses traditional management practices (e.g., cows housed in small brick barns) predominately located in the Northern Highlands but scattered in several governorates.

There are also small ruminant farms (i.e., flocks), generally, more scattered throughout Jordan than the dairy cattle farms. They are raised in both the Northern Highlands and the Badia region. The Northern Highlands region receives the highest amounts of rainfall in Jordan and is occupied by small herders who rely on extensive production systems and use rangelands in a constant search for grass and water (*1*). In contrast, Badia is an arid to semiarid region located in southern and eastern Jordan and occupied by nomadic or pastoralist Bedouin (*1*).

Materials and Methods

Setting

There is no animal vaccination program for Rift Valley fever (RVF) in Jordan. This study was designed as a cross-sectional study that covered all governorates of Jordan except Madaba and Aqaba. Several farms across each governorate were randomly selected for the study.

Serum Sample Collection

From each animal, 10 mL of blood was collected and transported to the laboratory on ice in a cooler on the same day of collection. At the laboratory, samples were registered in a logbook and the serum collected by centrifuging the blood samples at 3,000 rpm for 10 min. Samples were stored in sterile 1.5 mL screw-capped tubes at −20°C and later shipped to Viral Special Pathogens Branch, Centers for Disease Control and Prevention, Atlanta, Georgia, USA for laboratory testing.

Serology (Caprine/Bovine/Ovine)- Immunoglobulin G Detection for RVF and CCHF

Indirect enzyme-linked immunosorbent assays (ELISA) were used to measure immunoglobulin G (IgG) levels in collected serum samples at least twice per sample. For RVF, viral antigen (Gamma-irradiated cell lysate of E6 Vero cells infected with RVF) in PBS (1:1,000) was added to the top half of a 96-well plate, and mock antigen (Gamma-irradiated cell lysate of uninfected E6 Vero cells) in PBS (1:1000) was added to the bottom half and incubated overnight at 4°C. After 3 washes with wash buffer (0.01M PBS-0.1% Tween-20), test serum as well as positive and negative control serum were added at a final 1:100 dilution to the first rows of the top and bottom halves of the plate, followed by 4-fold serial dilution down each column. Plates were incubated at 37°C for 1 h and washed 3 times. Anti–sheep IgG conjugated to horseradish peroxidase (HRPO) (KPL Cat. No. 14–23–06, Seracare, https://www.seracare.com) was added to the whole plate and incubated at 37°C for 1 h, followed again by 3 washes. Substrate ABTS was added and incubated at 37°C for 30 min; in the presence of HRPO and hydrogen peroxide, a detectable color transformation occurs that is described by an optical density (OD) value proportional to the amount of IgG present. Positive samples are determined

using an adjusted OD generated by subtracting the negative antigen OD from the positive antigen OD and comparing against established criteria.

CCHF antibodies were detected as described previously (*4*,*5*). In brief, the 96-well plate was coated with anti-CCHF Hyperimmune Mouse Ascitic Fluids (HMAF) in PBS (1:1,000) and incubated overnight at 4°C. After 3 washes with wash buffer, CCHF viral antigen (Gamma-irradiated E6 Vero cells infected with CCHF) diluted in PBS (1:10) was added to the top half of the plate with mock antigen (Gamma-irradiated uninfected E6 Vero cells) in PBS (1:10) added to the bottom half. The subsequent steps and accompanying reagents and conditions are the same as described above for the RVF IgG ELISA with the exception of the conjugate, for which mouse anti-bovine IgG conjugated to HRPO (Catalog # MA5–16733; ThermoFisher, https://thermofisher.com) was used for cattle samples. All plates were read at 410 nm using a Biotek Powerwave reader with Gen 5 software (Biotek Instruments, https://www.biotek.com).

Data Management and Statistical Analyses

The criteria we used to call a sample positive for IgG were a sum OD >0.95 and IgG titer ≥1:400. Data were entered into Microsoft Excel spreadsheet. The overall seroprevalence for each species was calculated as the total number of serologically positive samples by the total number of all tested samples for that species. A herd was considered positive when ≥1 animal was seropositive. Herd-level seroprevalence was calculated by dividing the number of seropositive herds for each animal species by the tested herds for that species.

References

1. Obaidat MM, Al-Zyoud AA, Bani Salman AE, Davis MA. Antimicrobial use and resistance among commensal *Escherichia coli* and *Salmonella enterica* in rural Jordan small ruminant herds. Small Rumin Res. 2017;149:99–104. https://doi.org/10.1016/j.smallrumres.2017.01.014

2. Obaidat MM, Bani Salman AE, Davis MA, Roess AA. Major diseases, extensive misuse, and high antimicrobial resistance of *Escherichia coli* in large- and small-scale dairy cattle farms in Jordan. J Dairy Sci. 2018;101:2324–34. PubMed https://doi.org/10.3168/jds.2017-13665

3. Department of Statistics (DoS). Agriculture surveys: livestock production value and quantity. 2015 [cited 2020 Dec 16]. http://web.dos.gov.jo/sectors/economic/agriculture/agriculture-surveys

4. Khan AS, Maupin GO, Rollin PE, Noor AM, Shurie HHM, Shalabi AGA, et al. An outbreak of Crimean-Congo hemorrhagic fever in the United Arab Emirates, 1994–1995. Am J Trop Med Hyg. 1997;57:519–25. PubMed https://doi.org/10.4269/ajtmh.1997.57.519

5. Logan TM, Linthicum KJ, Moulton JR, Ksiazek TG. Antigen-capture enzyme-linked immunosorbent assay for detection and quantification of Crimean-Congo hemorrhagic fever virus in the tick, *Hyalomma truncatum*. J Virol Methods. 1993;42:33–44. PubMed https://doi.org/10.1016/0166-0934(93)90174-P
